# Supplementary material for: Complex Characterization of Germline Large Genomic Rearrangements of the BRCA1 and BRCA2 Genes in High-Risk Breast Cancer Patients—Novel Variants from a Large National Center
Source: Int J Mol Sci. 2020 Jun 30;21(13):4650. doi: 10.3390/ijms21134650 (PMC7370166; doi:10.3390/ijms21134650)
Supplement: Supplementary file 1 [file ijms-21-04650-s001.zip › Supplementary/Figure S1.pptx]

## Slide 1
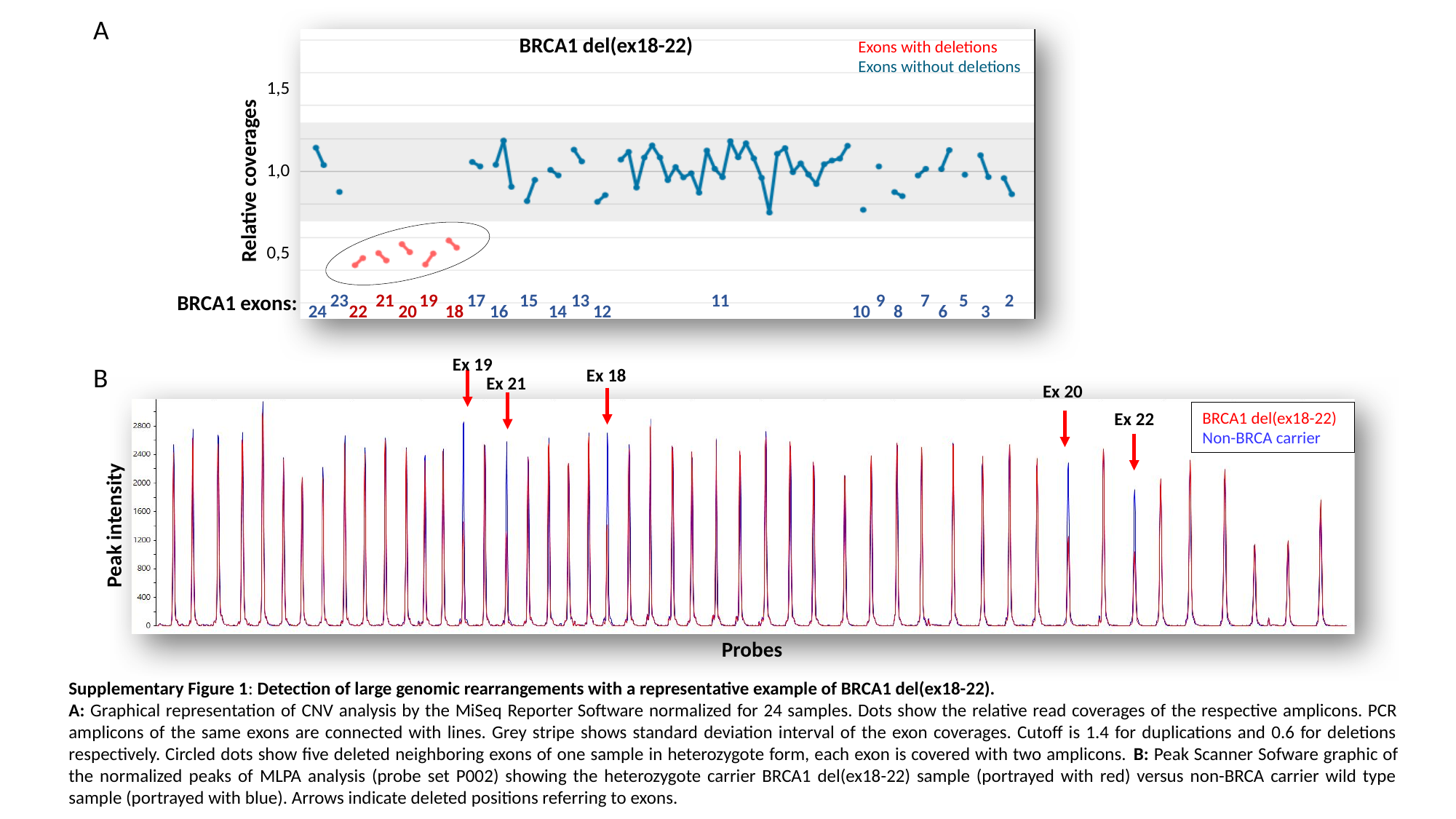

A
BRCA1 del(ex18-22)
Exons with deletions
Exons without deletions
1,5
Relative coverages
1,0
0,5
23
21
19
17
15
13
11
9
7
5
2
BRCA1 exons:
24
22
20
18
16
14
12
10
8
6
3
Ex 19
B
Ex 18
Ex 21
Ex 20
Ex 22
BRCA1 del(ex18-22)
Non-BRCA carrier
Peak intensity
Probes
Supplementary Figure 1: Detection of large genomic rearrangements with a representative example of BRCA1 del(ex18-22).
A: Graphical representation of CNV analysis by the MiSeq Reporter Software normalized for 24 samples. Dots show the relative read coverages of the respective amplicons. PCR amplicons of the same exons are connected with lines. Grey stripe shows standard deviation interval of the exon coverages. Cutoff is 1.4 for duplications and 0.6 for deletions respectively. Circled dots show five deleted neighboring exons of one sample in heterozygote form, each exon is covered with two amplicons. B: Peak Scanner Sofware graphic of the normalized peaks of MLPA analysis (probe set P002) showing the heterozygote carrier BRCA1 del(ex18-22) sample (portrayed with red) versus non-BRCA carrier wild type sample (portrayed with blue). Arrows indicate deleted positions referring to exons.
