# Supplementary material for: Complex Characterization of Germline Large Genomic Rearrangements of the BRCA1 and BRCA2 Genes in High-Risk Breast Cancer Patients—Novel Variants from a Large National Center
Source: Int J Mol Sci. 2020 Jun 30;21(13):4650. doi: 10.3390/ijms21134650 (PMC7370166; doi:10.3390/ijms21134650)
Supplement: Supplementary file 1 [file ijms-21-04650-s001.zip › Supplementary/Figure S2.pptx]

## Slide 1
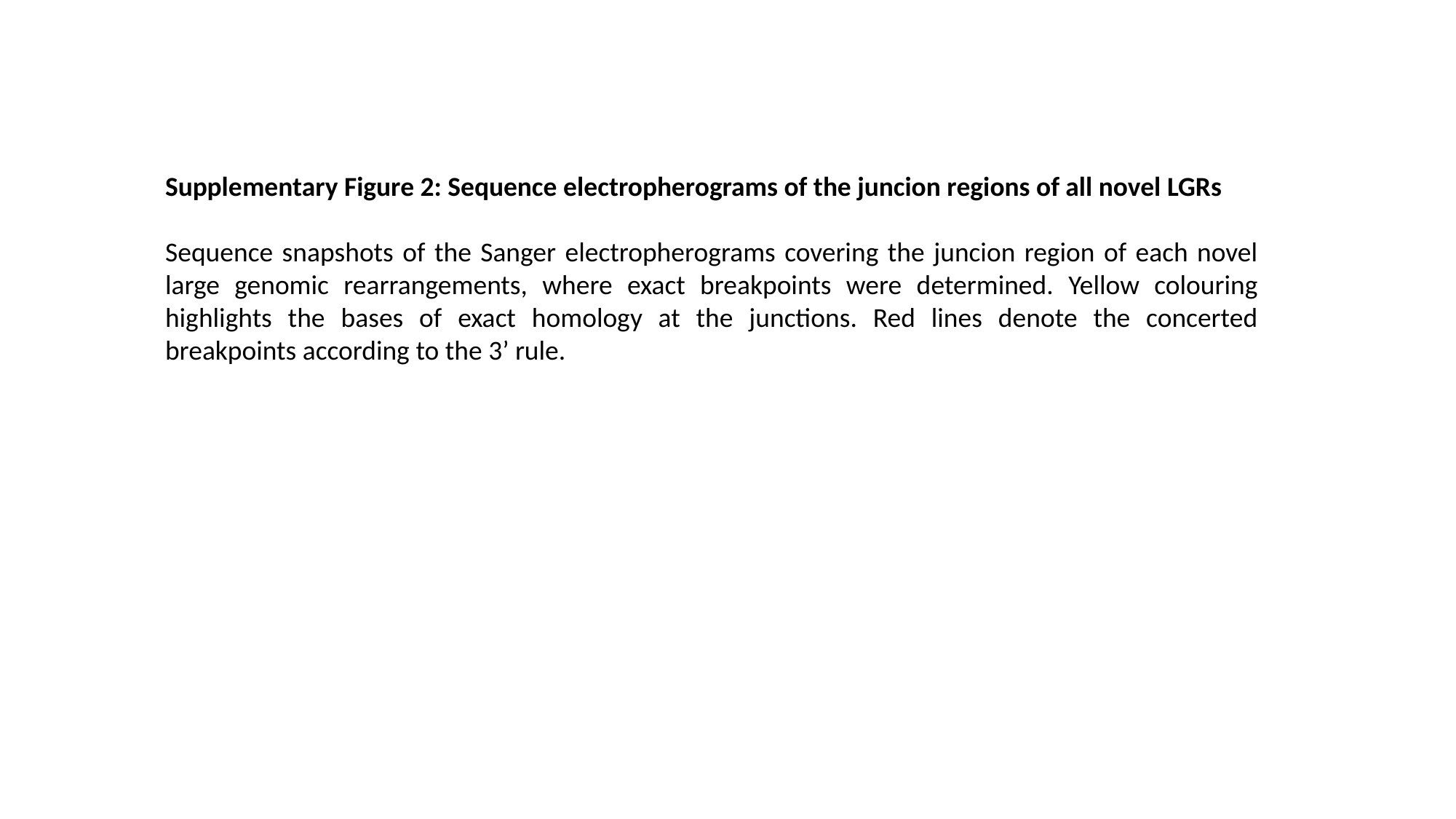

Supplementary Figure 2: Sequence electropherograms of the juncion regions of all novel LGRs
Sequence snapshots of the Sanger electropherograms covering the juncion region of each novel large genomic rearrangements, where exact breakpoints were determined. Yellow colouring highlights the bases of exact homology at the junctions. Red lines denote the concerted breakpoints according to the 3’ rule.

## Slide 2
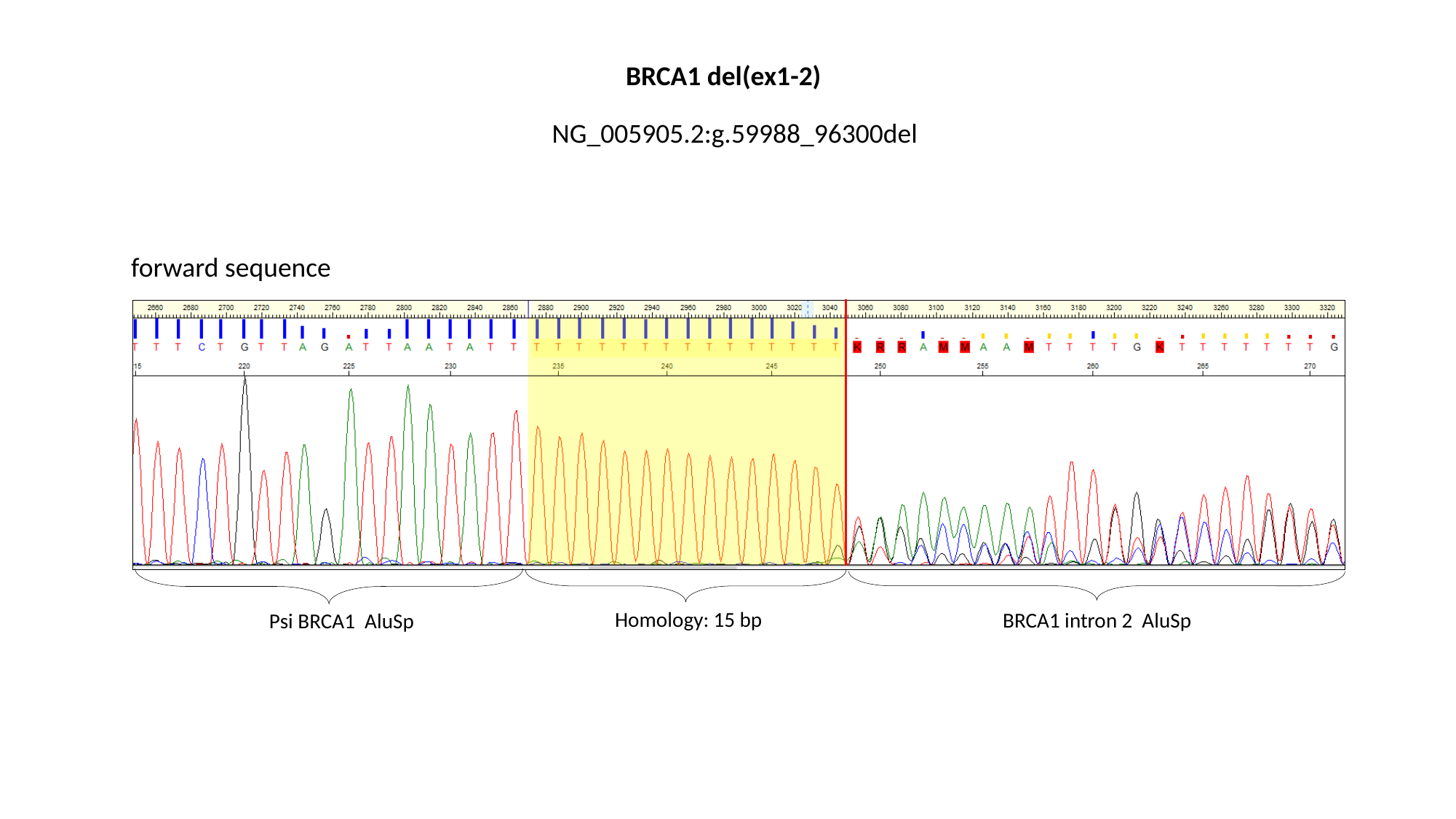

BRCA1 del(ex1-2)
NG_005905.2:g.59988_96300del
forward sequence
Homology: 15 bp
BRCA1 intron 2 AluSp
Psi BRCA1 AluSp

## Slide 3
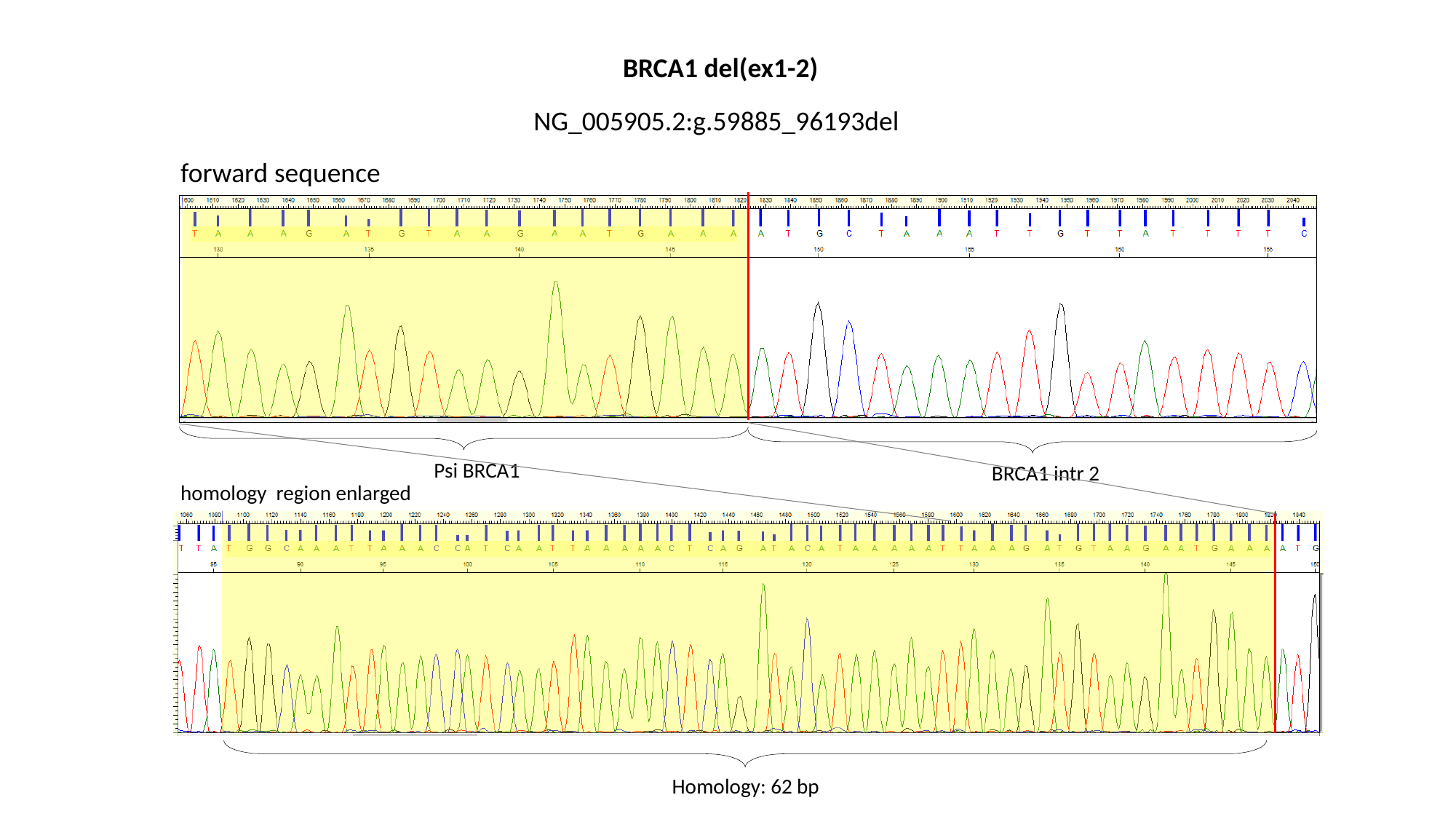

BRCA1 del(ex1-2)
NG_005905.2:g.59885_96193del
forward sequence
Psi BRCA1
BRCA1 intr 2
homology region enlarged
Homology: 62 bp

## Slide 4
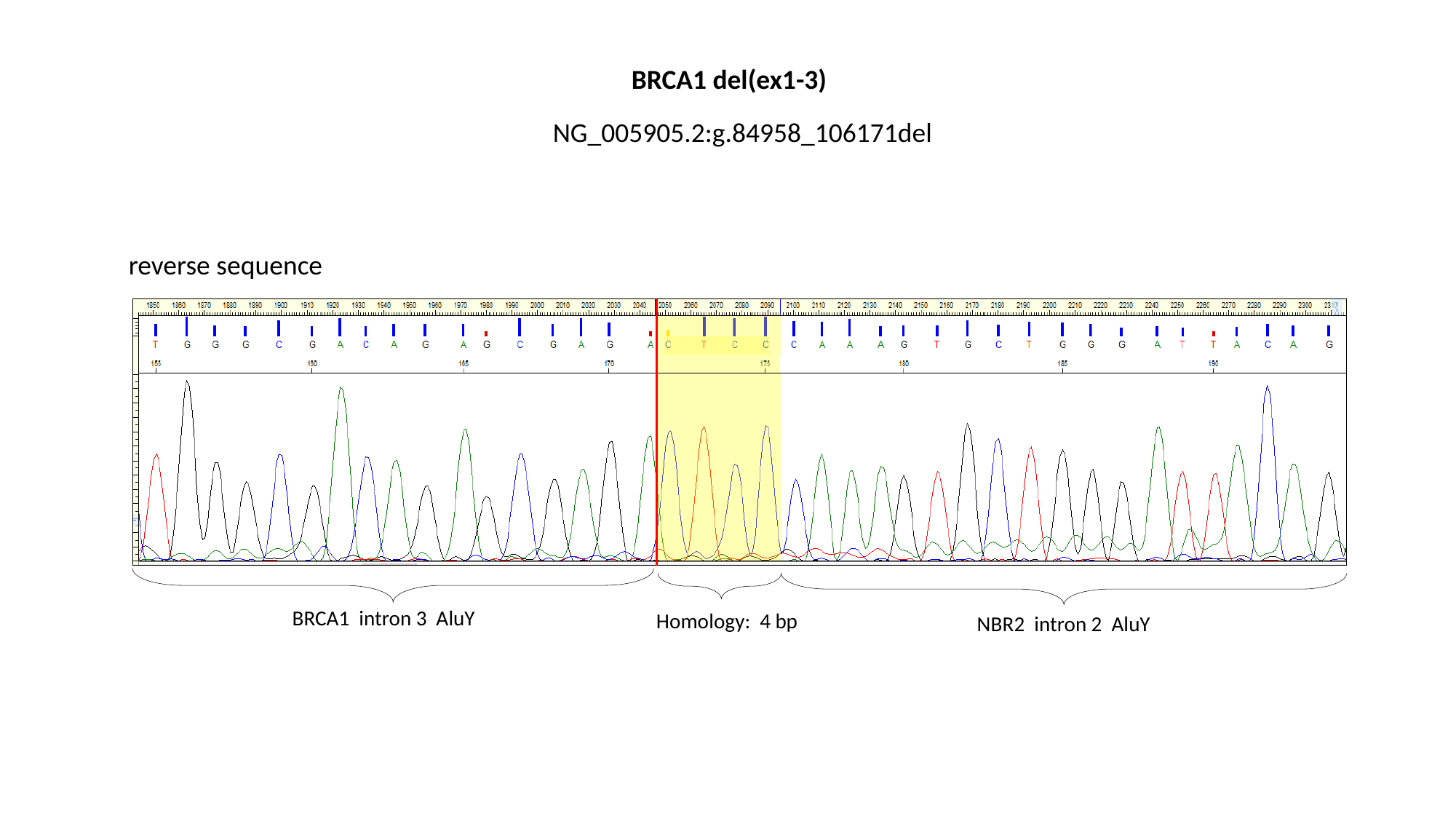

BRCA1 del(ex1-3)
NG_005905.2:g.84958_106171del
reverse sequence
BRCA1 intron 3 AluY
Homology: 4 bp
NBR2 intron 2 AluY

## Slide 5
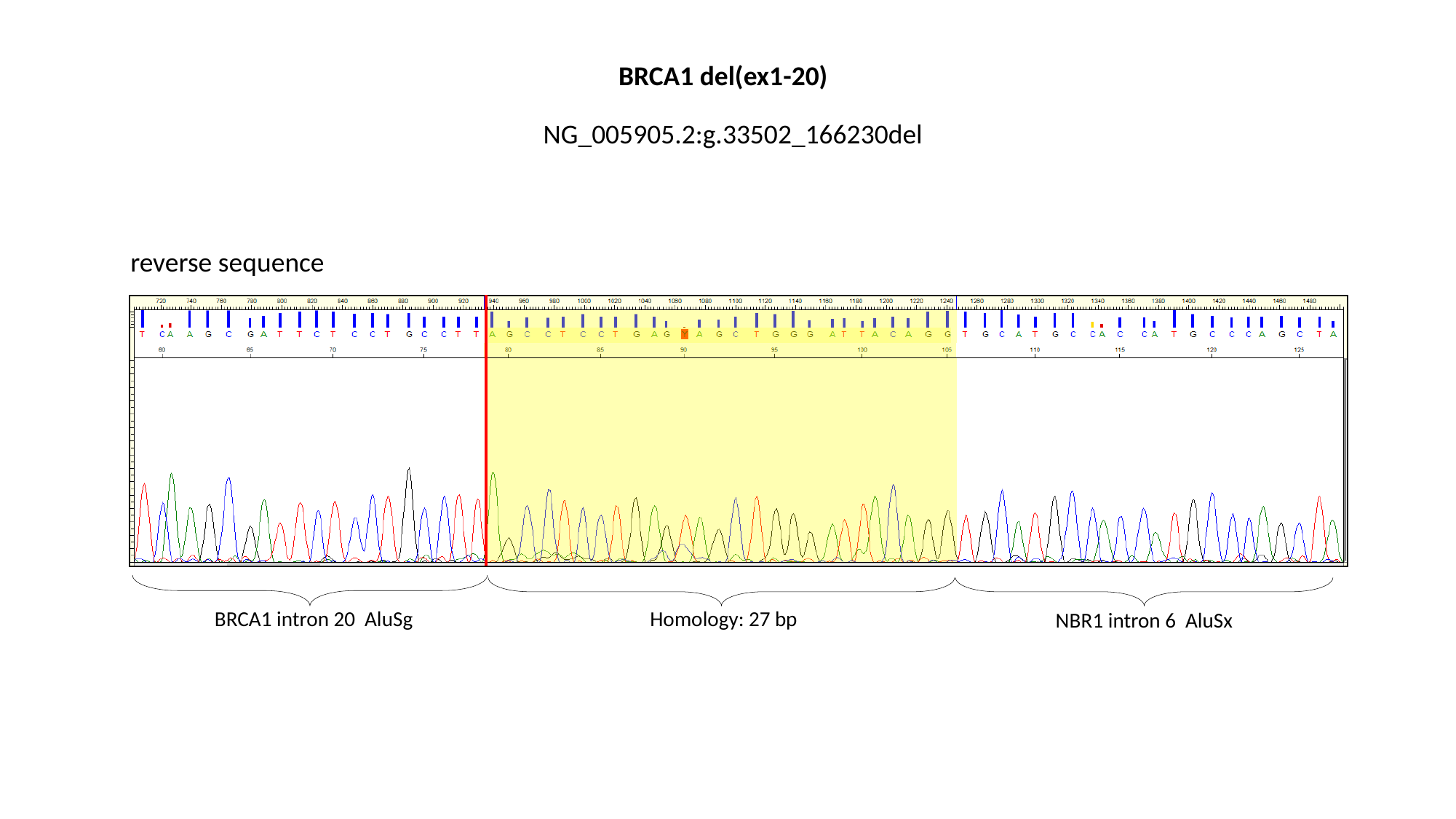

BRCA1 del(ex1-20)
NG_005905.2:g.33502_166230del
reverse sequence
BRCA1 intron 20 AluSg
Homology: 27 bp
NBR1 intron 6 AluSx

## Slide 6
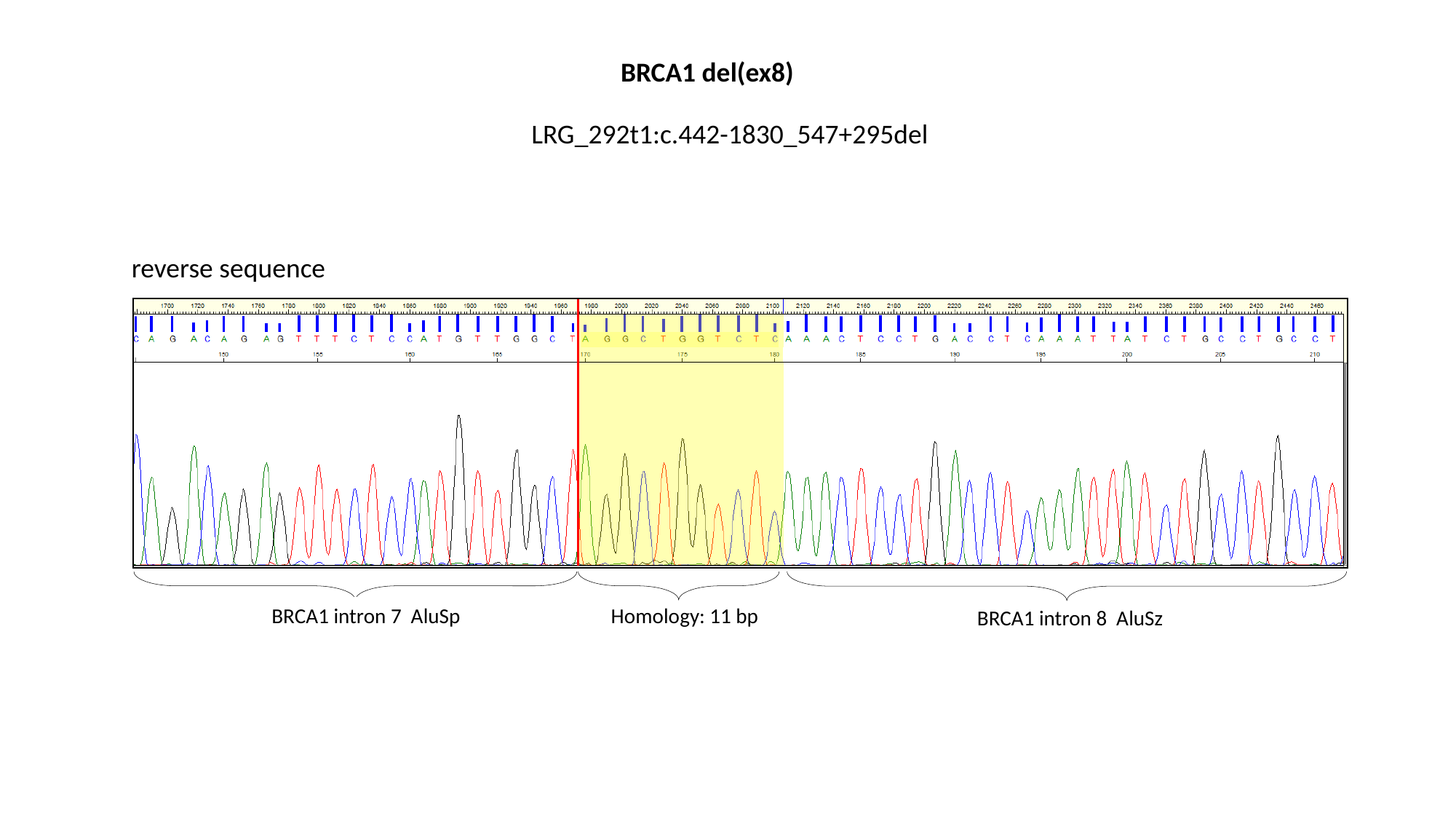

BRCA1 del(ex8)
LRG_292t1:c.442-1830_547+295del
reverse sequence
BRCA1 intron 7 AluSp
Homology: 11 bp
BRCA1 intron 8 AluSz

## Slide 7
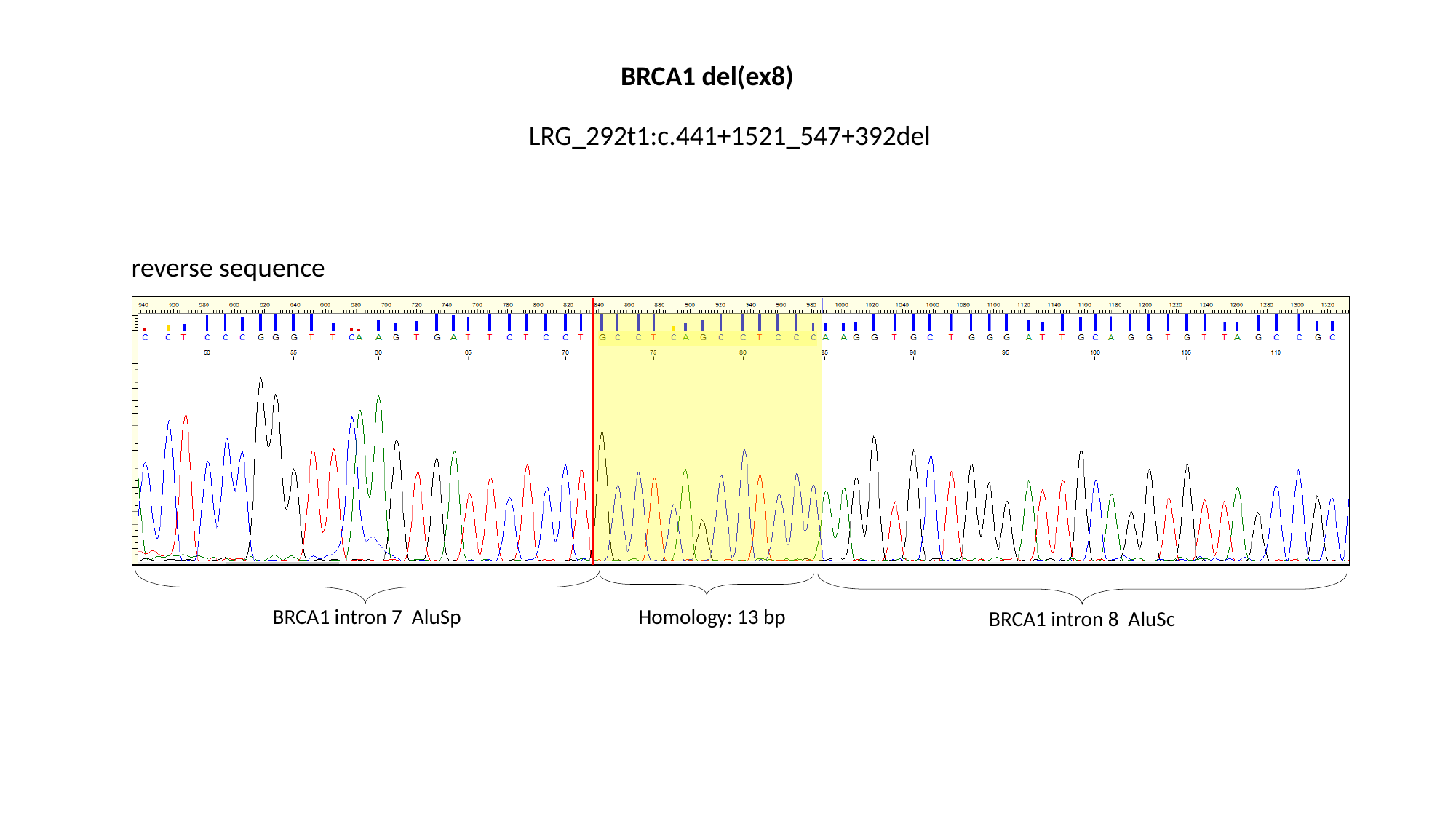

BRCA1 del(ex8)
LRG_292t1:c.441+1521_547+392del
reverse sequence
BRCA1 intron 7 AluSp
Homology: 13 bp
BRCA1 intron 8 AluSc

## Slide 8
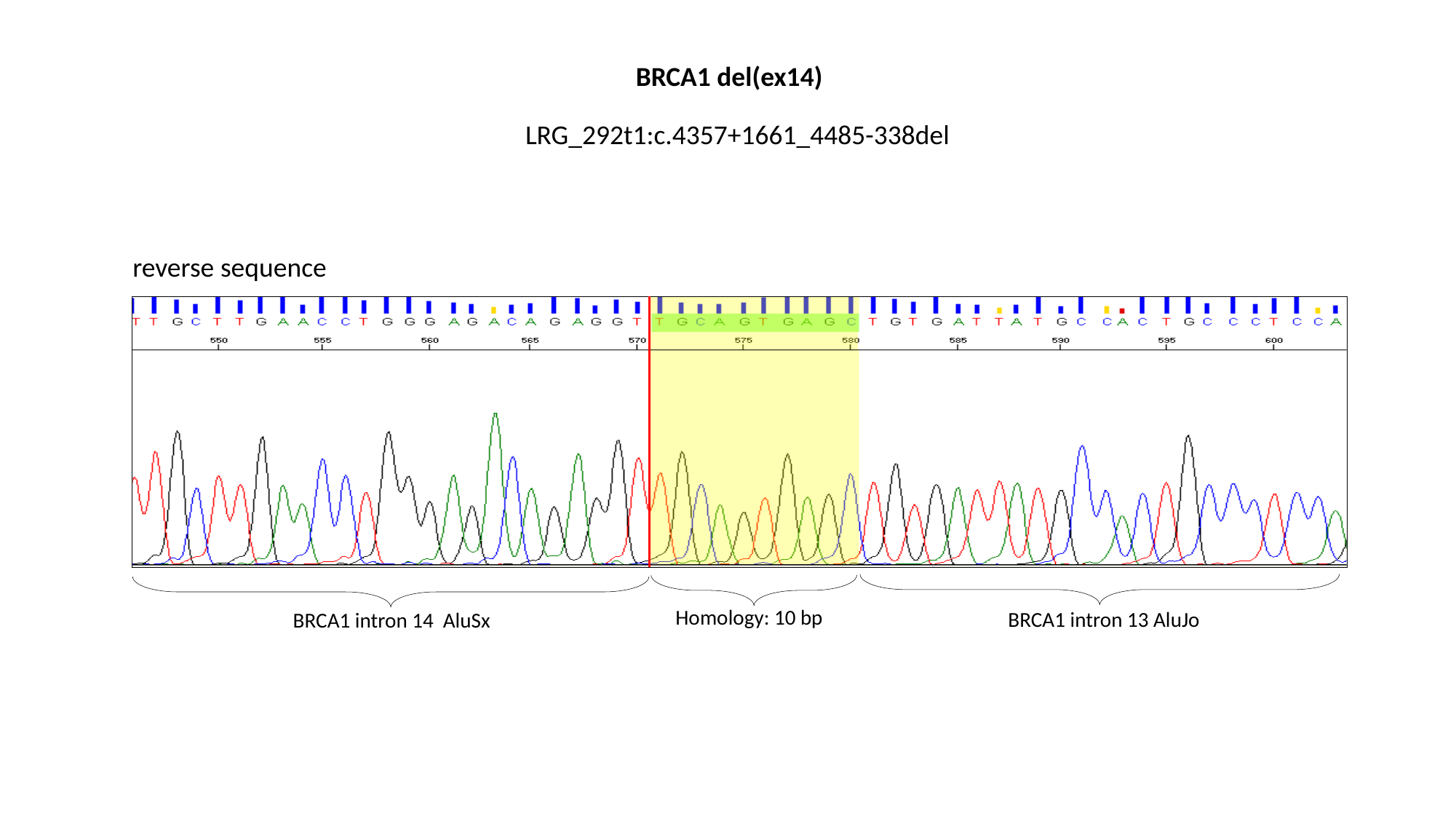

BRCA1 del(ex14)
LRG_292t1:c.4357+1661_4485-338del
reverse sequence
Homology: 10 bp
BRCA1 intron 13 AluJo
BRCA1 intron 14 AluSx

## Slide 9
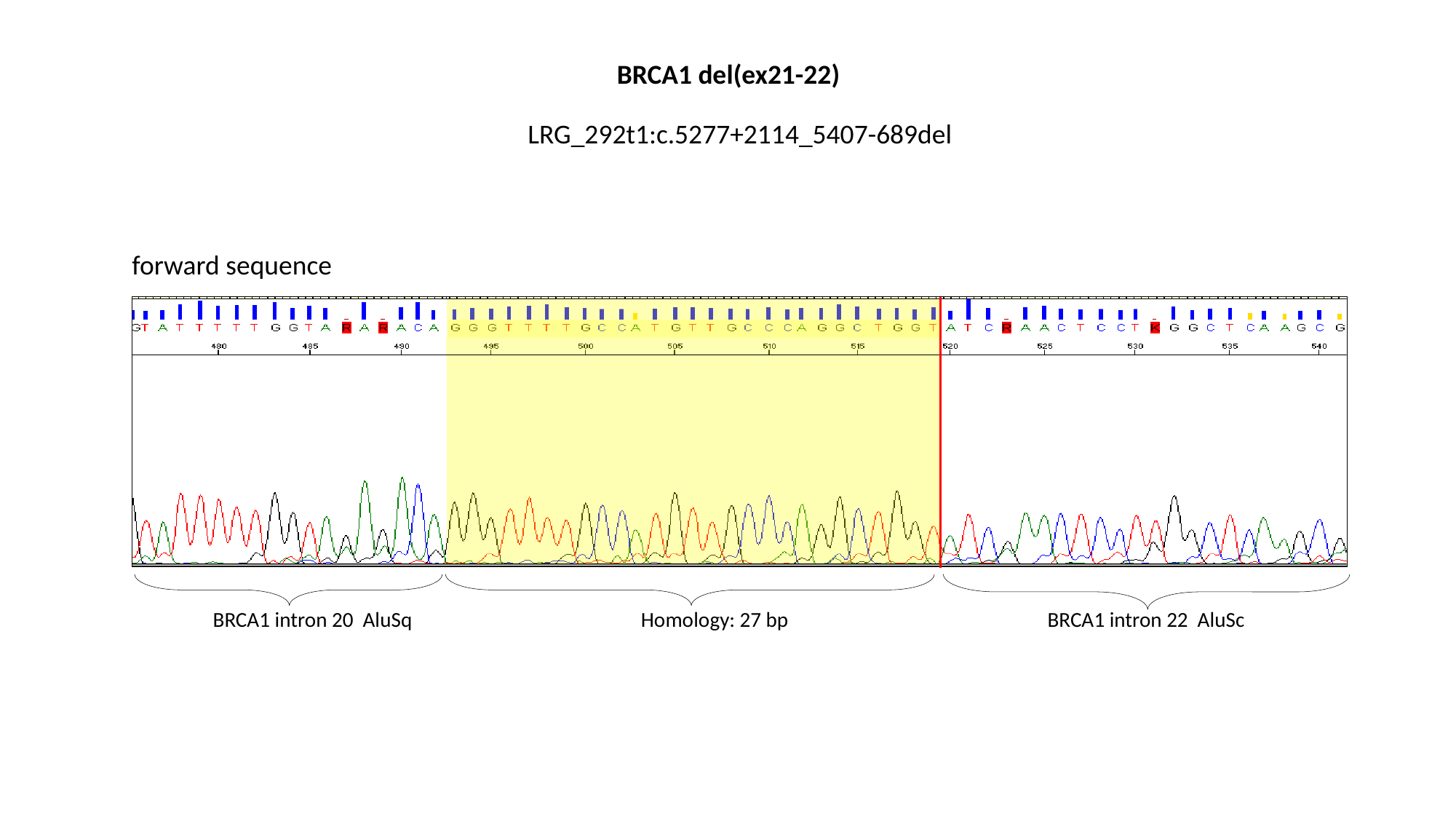

BRCA1 del(ex21-22)
LRG_292t1:c.5277+2114_5407-689del
forward sequence
BRCA1 intron 20 AluSq
Homology: 27 bp
BRCA1 intron 22 AluSc

## Slide 10
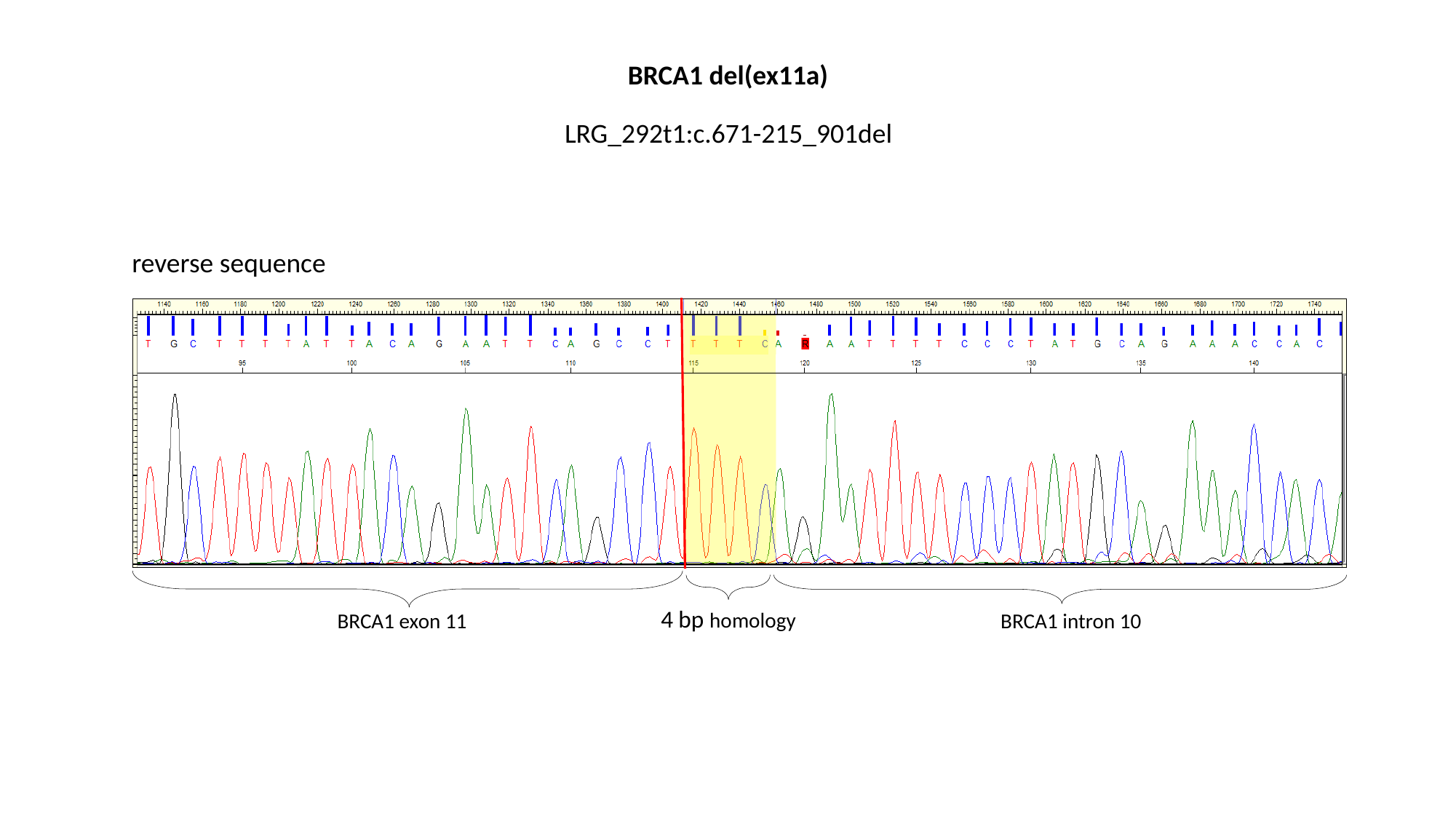

BRCA1 del(ex11a)
LRG_292t1:c.671-215_901del
reverse sequence
4 bp homology
BRCA1 exon 11
BRCA1 intron 10

## Slide 11
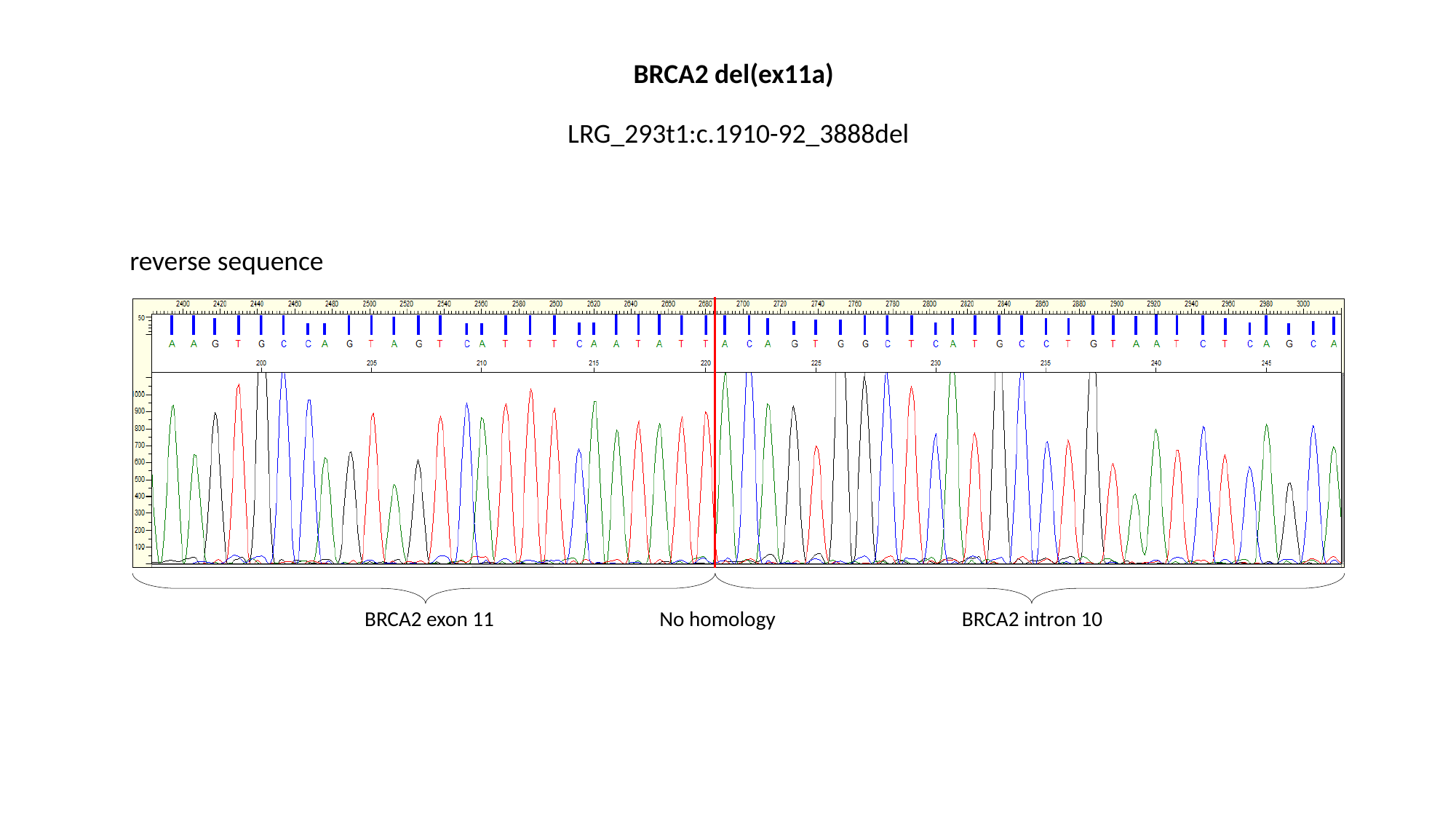

BRCA2 del(ex11a)
LRG_293t1:c.1910-92_3888del
reverse sequence
BRCA2 exon 11
No homology
BRCA2 intron 10
